# Rapid, label-free differentiation of benign and malignant gastric lesions using two-photon microscopy and quantitative collagen analysis

**DOI:** 10.1515/jtim-2026-0070

**Published:** 2026-08-24

**Authors:** Bo Ning, Xiaoqing Zhou, Kaiyue Guan, Biao Yan, Qianqian Chen, Jinping Li, Shenzhen Liu, Qun Shao, Lihua Sun, Sikun Zhang, Xinhua Ji, Dawei Li, Jinfeng Zang, Enqiang Linghu

**Affiliations:** Southeast University School of Medicine, Nanjing, Jiangsu Province, China; Department of Gastroenterology, the First Medical Center of PLA General Hospital, Beijing, China; Nankai University School of Medicine, Nankai University, Tianjin, China; The College of Future Technology, Peking University, Beijing, China; Department of Pathology, the First Medical Center of PLA General Hospital, Beijing, China

**Keywords:** two-photon imaging, gastric cancer, label-free diagnosis, collagen

## Abstract

**Background and Objectives:**

Previous investigations have explored two-photon microscopy (TPM) for differentiating normal and malignant lesions in the digestive tract. The aim of this study is to evaluate label-free TPM for rapid, *ex vivo* classification of fresh endoscopic human gastric tissues across the spectrum of neoplastic progression, identify quantitative imaging biomarkers, and establish a basis for near real-time diagnostics.

**Methods:**

Sixty fresh gastric samples (8 normal, 34 chronic inflammation/metaplasia, 10 atypical hyperplasia, 8 malignant lesions) from 32 patients undergoing routine endoscopic biopsy were imaged using TPM without fixation/staining, generating 60 mosaics and 122 3D images. Key morphological features were established and validated by two blinded pathologists. Normal, chronic inflammation/metaplasia lesions were classified into the low-risk group, while atypical hyperplasia and malignant lesions were assigned to the high-risk group. Second-harmonic generation (SHG) quantified collagen area percentage (Area%) and orientation dispersion (Dispersion) to differentiate low-risk group from high-risk group.

**Results:**

TPM provided high-resolution visualization of cellular/stromal features essential for pathology, comparable to histology but significantly faster. Blinded review showed substantial inter-reader agreement (Cohen's κ = 0.793) and 80.3% diagnostic accuracy across five tissue types. Quantitative analysis revealed high-risk lesions had significantly greater collagen Dispersion (*U* = 684, *P* < 0.001, *r* = −0.498) and higher Area% (*U* = 376, *P* < 0.001, *r* = −0.644) than low-risk lesions.

**Conclusions:**

TPM offers a robust, label-free, rapid method for *ex vivo* assessment of gastric biopsies, accurately differentiating lesions from normal to malignant lesions. Increased collagen Dispersion and Area% serve as objective biomarkers for distinguishing high-risk lesions. These findings support TPM's transformative potential for near real-time pathological evaluation, accelerating diagnostics, improving biopsy targeting, and enabling AI-assisted bedside/intraprocedural tools in gastric atypical hyperplasia and malignant lesions management.

## Introduction

Gastric cancer represents a significant global oncology burden, ranking fifth in incidence and fourth in cancer mortality worldwide in 2020.^[1]^ The clinical outcome for patients with gastric malignant lesions is critically dependent on the stage at diagnosis: early-stage disease, when detected and managed appropriately, is associated with a 5-year survival rate exceeding 90%,^[2]^ however, advanced-stage disease carries a dismal prognosis, with 5-year survival rates often falling below 30%.^[3, 4, 5, 6]^ This dramatic disparity underscores the urgent clinical need for improved strategies for early and accurate diagnosis to enhance patient survival and quality of life.^[7]^ The current cornerstone of gastric lesion diagnosis involves endoscopic biopsy followed by conventional histopathological examination.^[7,8]^ The current gold standard, endoscopic biopsy followed by histopathology, is time-consuming due to fixation, embedding, sectioning, and staining, creating diagnostic delays.

Two-photon microscopy (TPM) has emerged as a powerful optical imaging modality with substantial potential to revolutionize label-free histopathology.^[9,10]^ Employing near-infrared femtosecond laser excitation, TPM concurrently generates two-photon excited fluorescence (TPEF) from endogenous cellular fluorophores (*e.g*., Nicotinamide adenine dinucleotide [NADH], flavin adenine dinucleotide [FAD], reflecting cellular metabolism and morphology) and second-harmonic generation (SHG) signals predominantly from highly ordered, non-centrosymmetric protein structures such as fibrillar collagen within the extracellular matrix (ECM).^[10, 11, 12]^ This intrinsic dual-modality imaging provides rich, complementary information on cellular and stromal architecture at subcellular resolution, akin to traditional histology but without exogenous stains. TPM offers several compelling advantages crucial for clinical translation:^[13, 14, 15]^ (1) label-free operation, obviating the need for staining procedures; (2) inherent optical sectioning for 3D imaging of intact tissues; (3) enhanced tissue penetration depth due to reduced scattering of near-infrared light; and (4) minimized phototoxicity, enabling potential application to living tissues. These attributes position TPM as an exceptionally promising technology for real-time, *in situ* or rapid *ex vivo* assessment of fresh tissues, potentially surmounting the limitations of current endoscopic imaging resolution and the inherent delays of conventional pathology.

While previous investigations have explored TPM for differentiating normal and malignant lesions in the digestive tract,^[15,16]^ including initial studies in gastric tissue by Chen *et al*.^[17]^ He K *et al*. for T-staging,^[18]^ and Li *et al*. for early gastric cancer changes,^[19]^ significant gaps hinder clinical translation. Previous studies have focused on limited tissue types, often restricted to binary comparisons (normal *vs*. cancer), utilized relatively small fields-of-view, or lacked comprehensive validation across the full pathological spectrum of gastric lesion progression, which crucially includes precancerous stages like intestinal metaplasia and atypical hyperplasia.^[20, 21, 22]^ Furthermore, methodological challenges, such as ensuring precise co-localization between TPM images of fresh tissue and subsequent H&E-stained sections from the identical tissue region, have sometimes limited direct feature correlation and robust validation. The predominant use of longitudinal tissue sections in prior TPM studies also presents a translational hurdle, as cross-sectional imaging is more directly relevant for future *in vivo* endoscopic TPM applications.

This study was designed to rigorously address these limitations and propel the clinical applicability of TPM for comprehensive gastric lesion diagnosis. Our specific aims were: (1) to conduct extensive TPM imaging on five distinct and clinically critical tissue types (normal, chronic inflammation, intestinal metaplasia, atypical hyperplasia, and malignant lesions); (2) to establish and validate a TPM diagnostic atlas *via* blinded pathologist review; (3) to identify and quantitatively validate collagen architectural alterations using SHG imaging as objective biomarkers for differentiating benign/low-risk lesions from malignant lesions and atypical hyperplasia lesions; and (4) to implement and adhere to a methodology ensuring precise co-localization and correlation between TPM images and gold-standard H&E pathology. This comprehensive study aimed to establish a robust foundation for the clinical translation of TPM as a rapid and reliable diagnostic tool for gastric diseases. The findings are anticipated to facilitate future integration with artificial intelligence, potentially leading to next-generation diagnostic platforms that can significantly impact patient care pathways.

## Materials and methods

### Patient cohort and tissue collection

This prospective study included 32 patients who underwent upper gastrointestinal endoscopy and biopsy at the First Medical Center of the Chinese PLA General Hospital between July 2024 and February 2025. The study protocol was approved by the Institutional Review Board (IRB) of the Chinese PLA General Hospital (S2024-741-01), and was conducted in accordance with the Declaration of Helsinki. All patients provided written informed consent prior to participation.

A total of 60 fresh gastric mucosal specimens were collected immediately after endoscopic biopsy. Inclusion criteria for biopsy were: (1) endoscopic suspicion of malignancy requiring pathological confirmation; (2) presence of gastric ulcers; or (3) areas of erosion or inflammation of indeterminate nature requiring further pathological evaluation. Patients included were aged between 18 and 80 years, irrespective of gender, and capable of providing informed consent. Exclusion criteria included patients belonging to vulnerable populations (*e.g*., minors, pregnant women), recent use (within 7 days) of oral anticoagulant medication, presence of severe coagulation disorders, or clinical conditions posing a high risk of gastrointestinal perforation (*e.g*., active gastrointestinal bleeding, recent history of significant bleeding, or severe unhealed ulcers).

The 60 collected samples were subsequently classified by gold-standard histopathology as: 8 normal tissues, 34 tissues exhibiting chronic inflammation or intestinal metaplasia, 10 tissues diagnosed with atypical hyperplasia, and 8 malignant lesions. In the present study, the normal group and malignant group each consisted of 8 cases, accounting for 13.3% (8/60) respectively, and the samples exhibited a class imbalance characteristic. Class imbalance may potentially lead to a shift in diagnostic weight bias among different groups. However, it is important to clarify that such sample class imbalance is essentially one of the inherent limitations of prospective studies. Prospective studies adhere to the core principle of clinical real-world case cohort enrollment, and the sample distribution strictly follows the natural incidence pattern of clinical diseases. In clinical practice, most patients undergoing gastroscopy present with symptoms related to gastritis or gastric discomfort; biopsy procedures are only performed when suspected lesions (such as chronic inflammation and intestinal metaplasia) are observed under endoscopic visualization. As benign lesions, chronic inflammation and intestinal metaplasia not only have a significantly higher incidence rate than malignant lesions and atypical hyperplasia lesions among biopsied patients but also a much higher incidence rate than normal mucosal tissues. Consequently, the prospectively enrolled samples naturally exhibit a distribution feature characterized by a high proportion of chronic inflammation and intestinal metaplasia groups, and a low proportion of normal, malignant lesions and atypical hyperplasia lesions transformation groups. Accordingly, we conclude that such class imbalance accords with objective clinical and pathological laws and does not affect the final study outcomes.

### Sample preparation for TPM

Immediately following endoscopic retrieval, fresh gastric mucosal samples were carefully flattened onto standard glass microscope slides. A minimal amount of Dulbecco's Modified Eagle Medium (DMEM) tissue preservation solution was applied to maintain tissue viability and hydration without immersion. This preparation method allowed for direct TPM imaging of the fresh, unprocessed tissue within minutes of biopsy.

### TPM imaging protocol

A high-resolution inverted two-photon microscope system (Transcend Vivoscope) was used for all imaging. The excitation source was a fiber-based femtosecond laser set to an excitation wavelength of 780 nm. TPEF signals were detected in the 420-580 nm spectral range, and SHG signals were specifically collected in the 375-405 nm range using appropriate bandpass filters and photomultiplier tubes (PMTs). The laser power delivered to the sample under the objective (25× water immersion, NA 1.05, Olympus) was maintained at approximately 80 mW to ensure adequate signal generation while minimizing potential photobleaching or photodamage. A hierarchical imaging strategy was employed for comprehensive tissue assessment. (1) Large-field mosaic Imaging: initially, large-field 2D mosaic images (composed of a 10 × 10 grid of individual frames, each with a field of view of 300 × 300 μm, resulting in a total imaged area of approximately 3 mm × 3 mm) were acquired at a tissue depth of around 30 μm from the mucosal surface. Every large-field image stitching and scanning takes approximately 5-6 min per sample. (2) 3D z-stack Imaging: based on morphological features identified in the large-field mosaics, 2-3 representative regions of interest (ROIs) were selected for detailed three-dimensional (d) z-stack imaging. ROIs selected per mosaic based on morphology, imaged from surface to about 100 μm depth (122 3D images total). SNR optimized (10 scans/frame, PMT gain = 0.6). Figures illustrating sample processing and the imaging workflow, corresponding to Figure 1, would be included here. Every 3D SHG image acquisition takes approximately 2-3 min.

**Figure 1:**
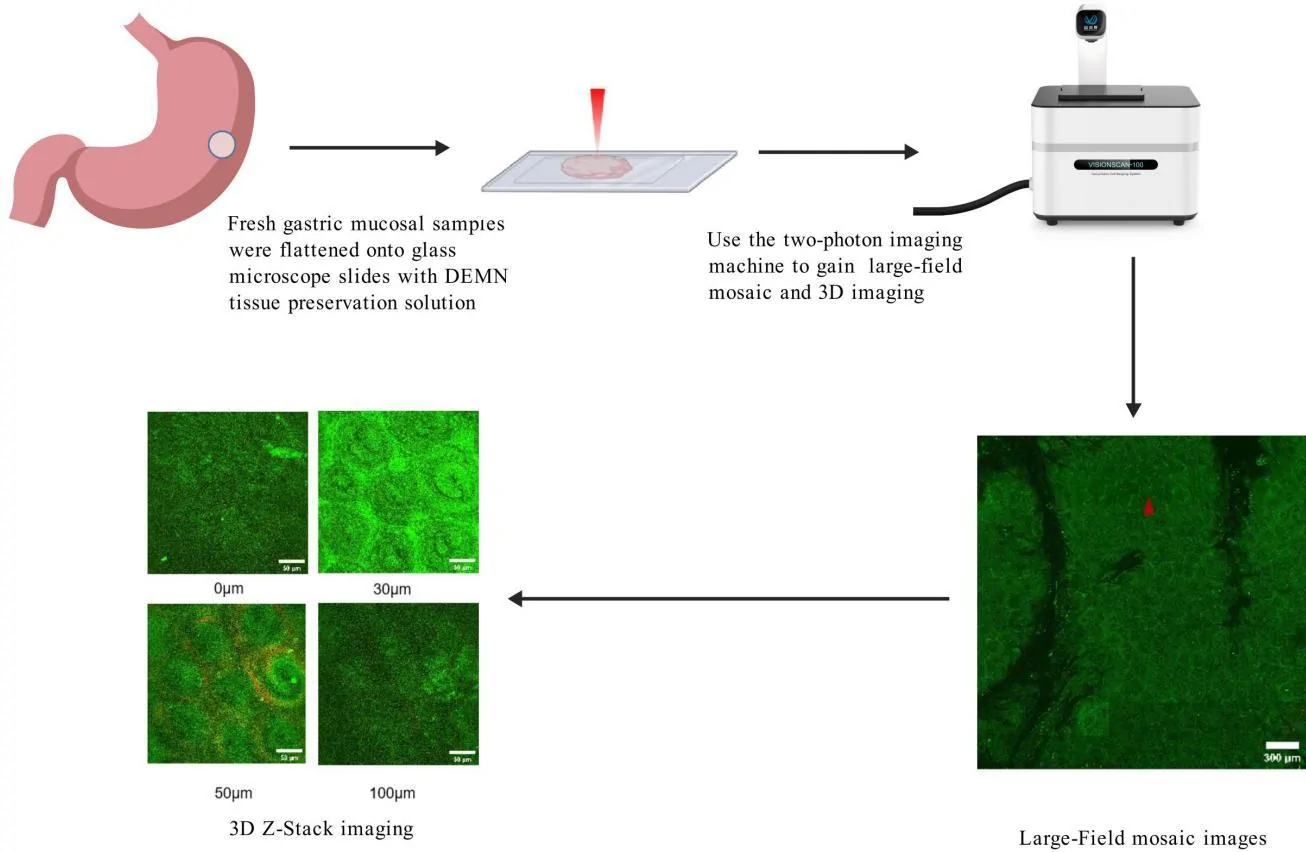
Processing of fresh isolated tissue and process of two-photon imaging of fresh ex vivo tissue, the red arrow indicates the region selected for 3D imaging within the wide-field view.

### Histopathological correlation and gold standard diagnosis

Following TPM imaging, the intact tissue specimen on the glass slide was immediately immersed in 10% neutral buffered formalin for fixation. The fixed tissues were then processed according to standard histological procedures. To ensure precise spatial correlation between the TPM-imaged regions and the subsequent H&E-stained sections, a meticulous cross-sectioning protocol was adopted. After initial coarse trimming to approach the depth of TPM imaging, serial sections (each 3-5 μm thick) were cut. Approximately 20 consecutive sections were collected, with the understanding that the TPM imaging depth (up to 100 μm, with key features often observed around 30-80 μm) would be encompassed within these serial sections. The H&E-stained slides were then carefully reviewed alongside the corresponding TPM images. This diagnosis was performed by a senior pathologist experienced in gastrointestinal pathology.

### Establishment of TPM diagnostic criteria and blinded pathological review

TPM features (TPEF/SHG) systematically correlated with H&E for all 5 tissue types, documenting glandular architecture, epithelial cellular features, and stromal collagen organization. (This atlas would be summarized in a table, corresponding to Table 1, and illustrated by representative TPM images for each category, corresponding to Figures 2-5 in the original manuscript).

**Figure 2:**
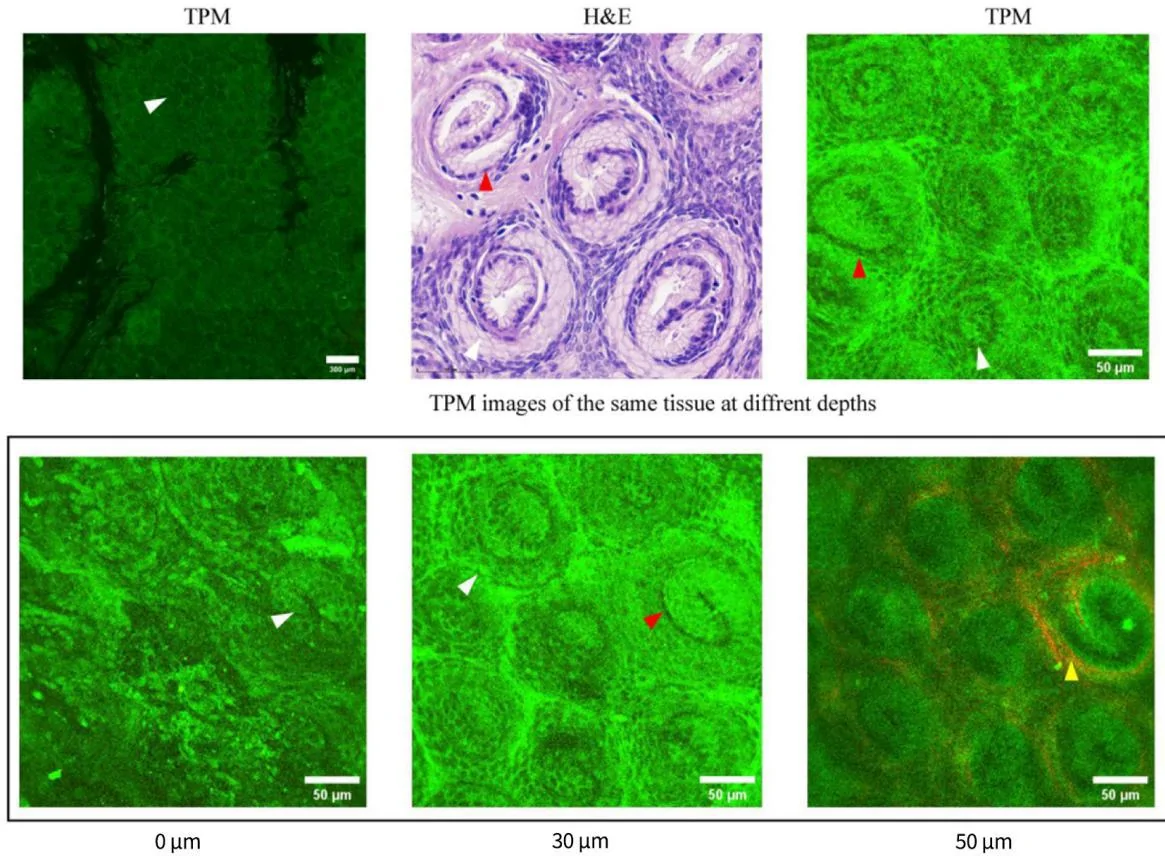
Representative images of normal tissue. White arrows represent the openings of gastric pits, red arrows represent cell nuclei. It can be observed that collagen fibers are almost undetectable between 0-30 μm. However, at 50 μm, distinct red collagen fibers, become clearly visible, evenly surrounding the glands (yellow arrows) in a honeycomb pattern. TPM: two-photon microscopy.

**Figure 3:**
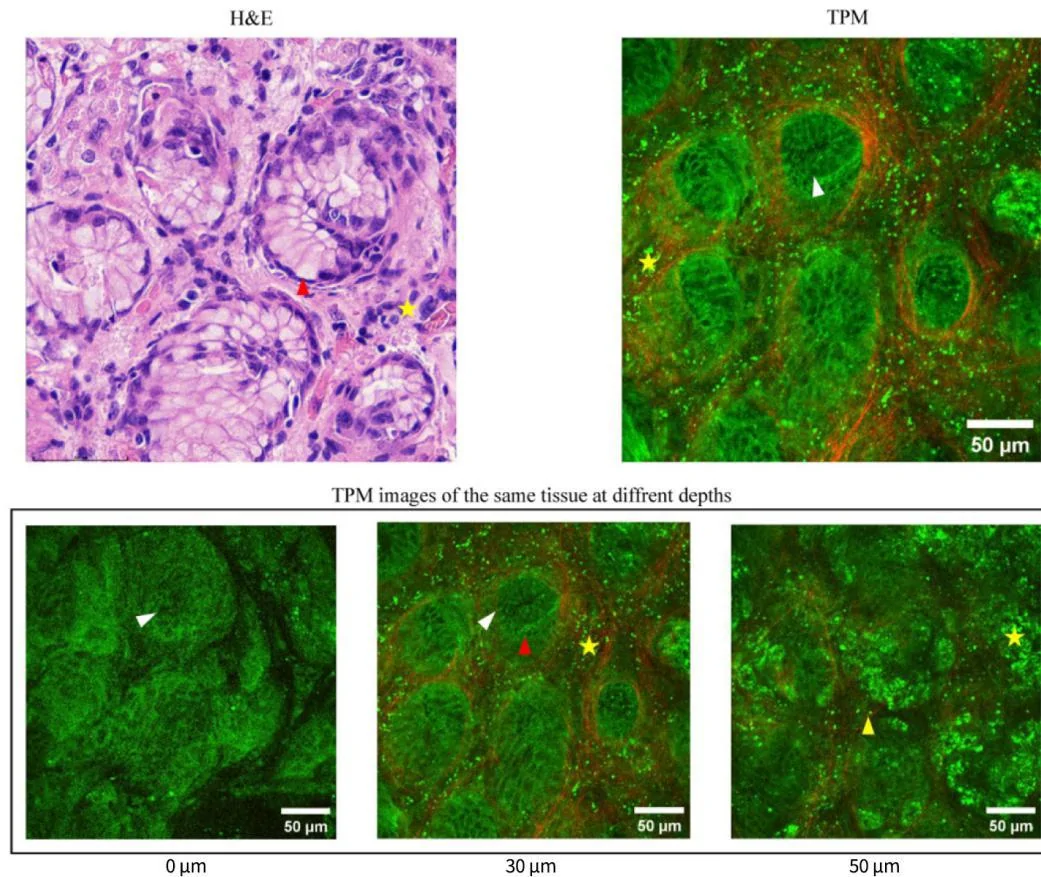
Representative images of chronic inflammatory tissue. White arrows represent the openings of gastric pits; red arrows represent cell nuclei; yellow arrow represents collagen fibers; and yellow stars represent plasma cells. TPM: two-photon microscopy.

**Figure 4:**
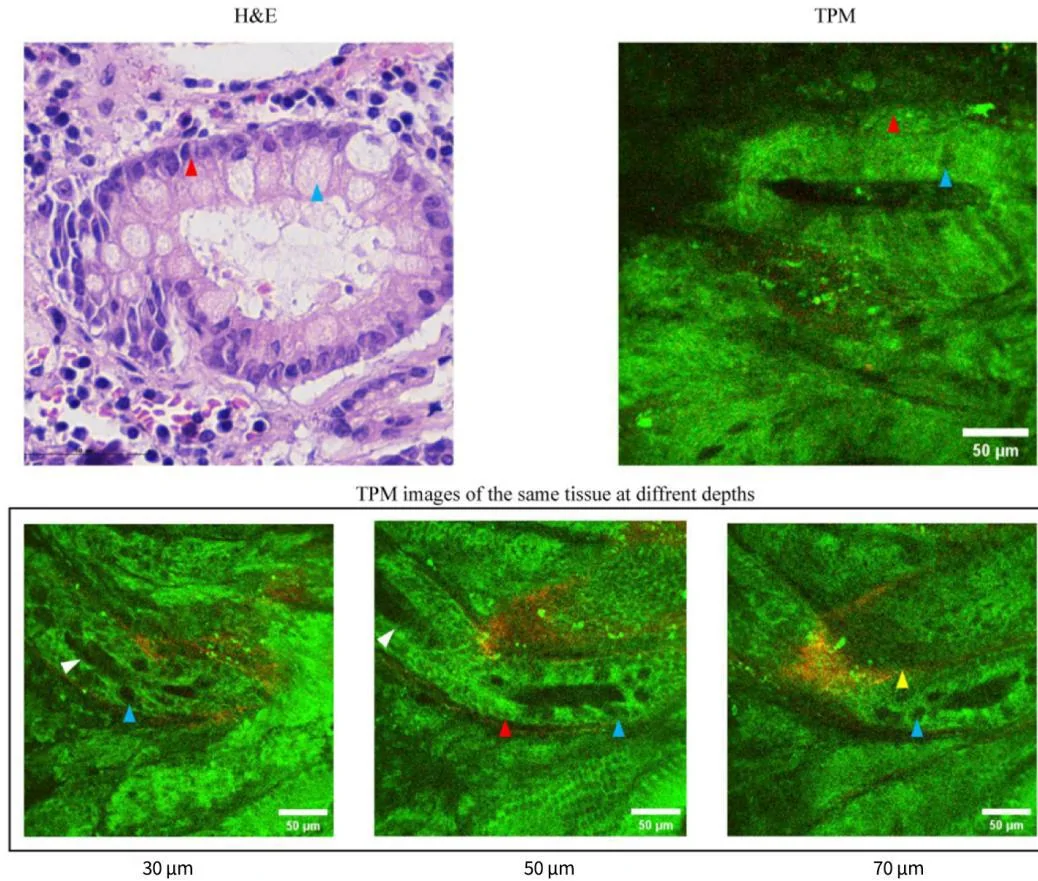
Representative two-photon images of intestinalized tissue. White arrows represent the openings of gastric pits; red arrows represent nuclei; yellow arrows represent collagen fibers; and blue arrows represent goblet cells. TPM: two-photon microscopy.

**Figure 5:**
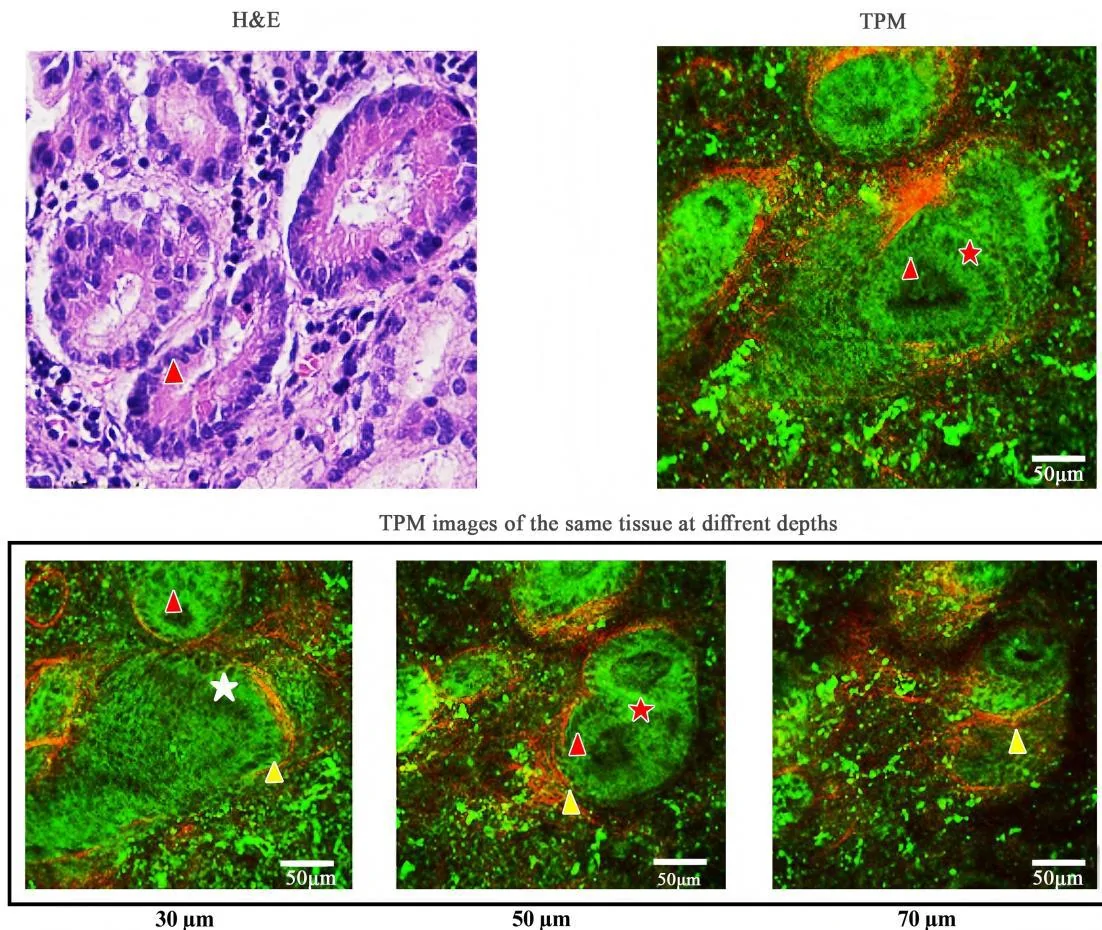
Representative two-photon images of atypical hyperplasia of gastric mucosa. Red arrows represent cell nuclei; yellow arrows represent collagen fibers; red stars represent atypical glands. TPM: two-photon microscopy.

**Table 1:** Key TPM pathological features of gastric tissues

Tissue Type	Glands	Epithelial cells (TPEF)	Collagen fibers (SHG)
Normal tissue	Regular size and shape, neatly arranged, distinct lumens	Single layer, columnar; nuclei small, basal, dark; cytoplasm strong, uniform granular fluorescence; low nucleocytoplasmic ratio	Fine, organized, honeycomb-like network uniformly surrounding glands; visible around 50 μm depth
Inflammatory tissue	Glands are generally regular, may be spaced apart by stromal infiltrate	Similar to normal, scattered bright TPEF spots from inflammatory cells in stroma, low nucleocytoplasmic ratio	Often thickened compared to normal, but generally maintains regular per glandular arrangement; may appear denser
Intestinal metaplasia	Glandular architecture is often irregular; presence of metaplastic glands	Goblet cells present (dark, mucus-filled vacuoles with weak TPEF); other epithelial cells may show altered morphology; a weak fluorescent ring may be present around the goblet cell nucleus	Often reduced in quantity, appearing sparse or fragmented around metaplastic glands
Atypical hyperplasia	Irregular gland size, shape, and spacing; crowding, back-to-back or cribriform patterns	Nuclei enlarged, pleomorphic, hyperchromatic-equivalent (increased/irregular TPEF); increased nucleocytoplasmic ratio; pseudo stratification, loss of polarity	Thickened, disorganized, fragmented, and irregularly distributed; overall per glandular pattern may be partially retained but distorted
Malignant lesions	Loss of normal glandular architecture; replaced by infiltrative nests, cords, or sheets	Marked cellular and nuclear pleomorphism; high nucleocytoplasmic ratio; irregular/coarse nuclear TPEF; loss of cell cohesion	Abundant, dense, highly disorganized, thick bundles (desmoplasia); infiltrating between tumor cells/nests; chaotic arrangement

Detailing TPM features for normal, inflammatory, intestinal metaplasia, atypical hyperplasia, and malignant lesions across glands, epithelial cells, and collagen fibers. TPM: two-photon microscopy; TPEF, two-photon excited fluorescence; SHG, second-harmonic generation.

To validate the utility of these established TPM features, the complete set of 122 3D TPM image stacks was anonymized, randomized, and presented to two independent pathologists for a blinded diagnostic review. These pathologists were tasked with classifying each image into one of the five categories. The consensus H&E diagnosis from the senior pathologist served as the gold standard against which the TPM-based diagnoses were compared.

### Quantitative collagen analysis

Quantitative analysis of collagen architecture was performed on the SHG channel images from the 3D TPM stacks using ImageJ software (v1.54p, National Institutes of Health, USA). For this analysis, the TPM images were categorized into two groups: group A (low-risk lesions—comprising normal, inflammatory, and intestinal metaplasia tissues; *n* = 78 images) and group B (low-risk lesions—comprising atypical hyperplasia and malignant lesions; *n* = 44 images). Two specific quantitative parameters were extracted from the SHG images. (1) Collagen fiber area%: this was calculated as the percentage of the total tissue area in the SHG image occupied by collagen signal above a defined intensity threshold, providing a measure of relative collagen content. (2) Collagen fiber orientation dispersion (Dispersion): this parameter was determined using a Fourier transform-based orientation analysis plugin in ImageJ (*e.g*., orientation]). It quantifies the heterogeneity or randomness of collagen fiber alignment within the ROI. Higher dispersion values indicate more disordered and less uniformly aligned collagen fibers.

### Statistical analysis

All statistical analyses were performed using SPSS software (version 25.0, IBM Corp., Armonk, NY, USA). Inter-observer agreement for the blinded TPM image diagnosis between the two pathologists was assessed using Cohen's Kappa (κ) coefficient. Diagnostic performance metrics, including accuracy, sensitivity, and specificity, were calculated for each pathologist and for each tissue type, using the gold-standard H&E diagnosis as the reference.

For the quantitative collagen data (Area% and Dispersion), normality of distribution was assessed using the Shapiro-Wilk test. As the data were found to not be normally distributed, they were expressed as Median (interquartile range [IQR]: Q1-Q3). The Mann-Whitney *U* test was employed for intergroup comparisons of Area% and Dispersion between Group A and Group B. Outliers were systematically identified using box plots and the 3*σ* rule; however, all data points were retained for analysis to maintain the integrity of the dataset.

A *P*-value < 0.05 was considered statistically significant for all tests. For significant Mann-Whitney *U* test results, the effect size (*r*) was calculated as $Z/\sqrt{N}$, where *Z* is the Z-statistic and *N* is the total number of observations.

## Results

### TPM morphological features across gastric tissue types

TPM imaging visualized distinct morphological characteristics for each of the five gastric tissue types studied. These features, derived from both TPEF and SHG signals, allowed for qualitative differentiation critical for pathological interpretation.

Normal gastric mucosa: characterized by regularly arranged gastric pits and glands lined by uniform epithelial cells with strong cytoplasmic TPEF. Nuclei were typically small, basal, and appeared as dark regions. SHG signals revealed well-organized, honeycomb-like collagen fibers in the lamina propria surrounding the glands, indicating structural integrity (Figure 2).

Chronic inflammation: showed preserved glandular architecture but with increased stromal cellularity (inflammatory infiltrate, often appearing as bright TPEF spots from metabolically active immune cells). Collagen fibers (SHG) were often thickened compared to normal tissue but generally maintained their organization around glands (Figure 3).

Intestinal metaplasia: identified by the presence of goblet cells, which appeared as dark, mucus-filled vacuoles with weak TPEF signals, within metaplastic glands. Glandular architecture could be somewhat irregular. Collagen fibers were often reduced or more sparsely arranged, reflecting altered tissue composition (Figure 4).

Atypical hyperplasia: displayed significant glandular distortion, including irregular gland size and shape, crowding, and occasionally back-to-back or cribriform patterns. Epithelial cells exhibited nuclear atypia (enlarged, pleomorphic, hyperchromatic-equivalent nuclei with increased TPEF signal in the nuclear region) and an increased nucleocytoplasmic ratio, key indicators of pre-malignant change. Collagen fibers were typically thickened, disorganized, and irregularly distributed, signifying early stromal remodeling (Figure 5).

Malignant lesions: characterized by overt loss of normal glandular structures, replaced by infiltrating nests, cords, or sheets of highly atypical tumor cells. Cells showed marked pleomorphism, high nucleocytoplasmic ratios, and irregular nuclear TPEF. SHG imaging revealed abundant, dense, and highly disorganized collagen bundles (desmoplasia) surrounding and infiltrating tumor cell clusters, a hallmark of invasive malignancy (Figure 6).

**Figure 6:**
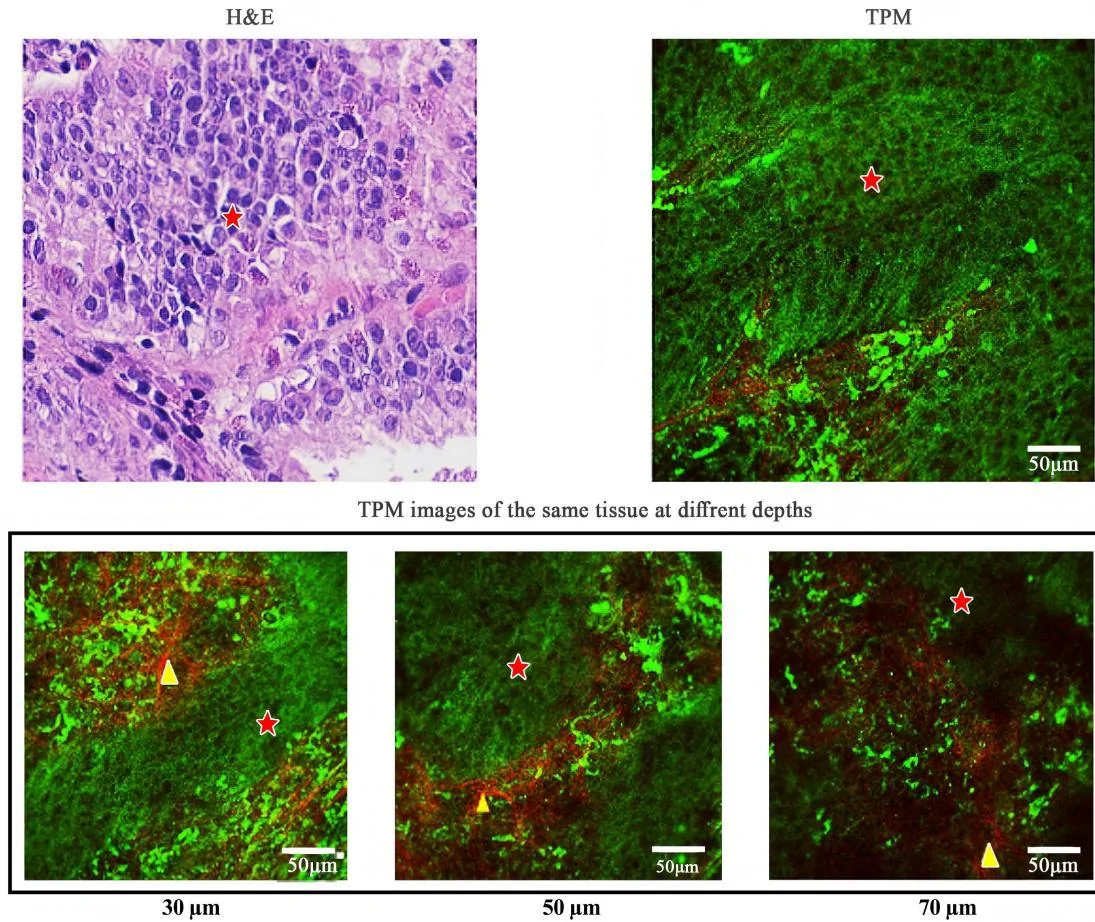
Representative two-photon images of malignant lesions. Red stars represent cancer cells; yellow arrows represent collagen fibers. TPM: two-photon microscopy.

A summary of these key distinguishing TPM features, forming the basis of a diagnostic atlas, is provided in Table 1.

### Blinded diagnostic performance using TPM images

The 122 anonymized 3D TPM image stacks were independently reviewed by two pathologists. Diagnostic indicators are calculated per specimen, as our ROIs are divided according to the regions of characteristic representative tissue samples. Before diagnosis, both received systematic training on TPM imaging principles and typical features of gastric lesions (normal, chronic inflammation, metaplasia, dysplasia, and malignancy).

A standardized diagnostic criterion defining the five lesion categories was strictly applied to ensure diagnostic objectivity and consistency. The two senior gastrointestinal pathologists underwent a one-week standardized training program focused on two-photon microscopic features of gastric lesions. During the entire reading process, all clinical information, H&E staining results, and pathological diagnoses were completely masked. Any inconsistent judgments were resolved through joint consultation and consensus discussion with a third senior pathologist until a unanimous conclusion was reached. The inter-observer agreement for classifying the images into one of the five categories was excellent, with an overall Cohen's Kappa value of 0.793 (95% confidence interval [95% CI] [0.704-0.882], *P* < 0.001), indicating the robustness and reproducibility of the TPM-based diagnostic criteria.

The overall diagnostic accuracy, when compared to the gold-standard H&E diagnosis, was 81.9% (100/122) for Pathologist 1 and 78.7% (96/122) for Pathologist 2, yielding a strong mean accuracy of 80.3%. Diagnostic performance varied by tissue type, providing insights into the strengths and current challenges of TPM diagnosis (representative plots in Figure 7 with error bars). (1) Normal tissue: mean accuracy 98.36%; mean sensitivity 90.91%; mean specificity 99.10%. (2) Inflammatory tissue: mean accuracy 84.43%; mean sensitivity 87.00%; mean specificity 82.64%. (3) Intestinal metaplasia: mean accuracy 93.44%; mean sensitivity 79.41%; mean specificity 95.71%. (4) Atypical hyperplasia: mean accuracy 86.89%; mean sensitivity 64.52%; mean specificity 94.51%. (5) Malignant lesions: mean accuracy 97.54%; mean sensitivity 84.62%; mean specificity 99.08%.

**Figure 7:**
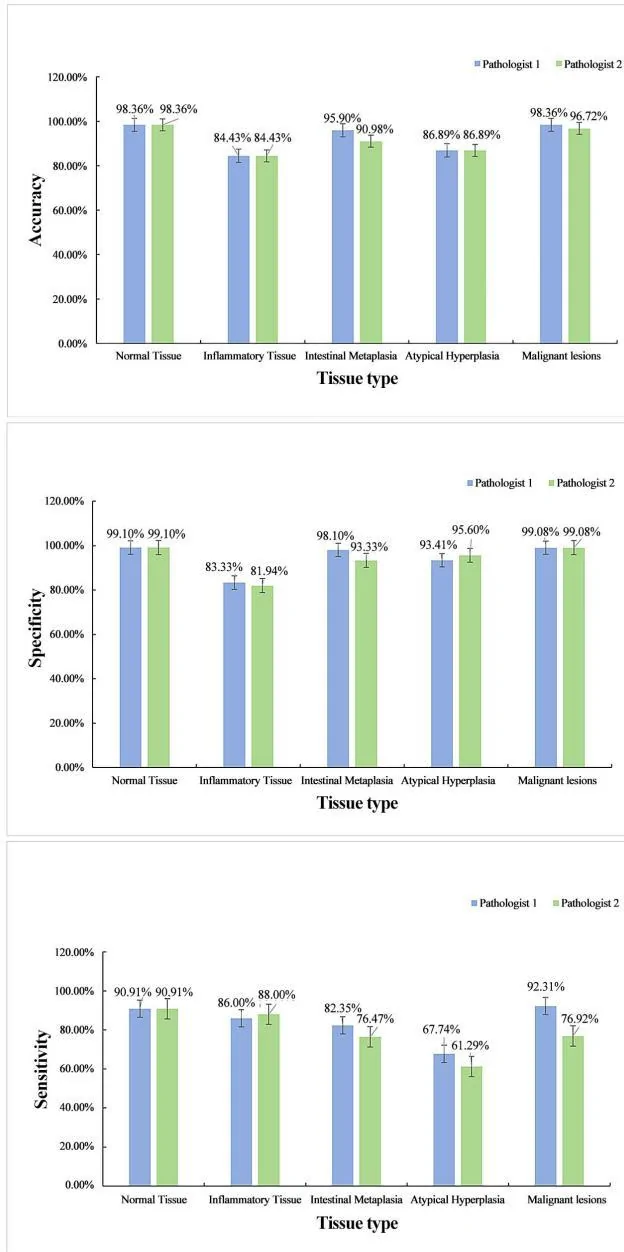
Diagnostic accuracy, sensitivity, and specificity of two pathologists for different tissue types, with error bars representing SEM. SEM: the standard error of the mean.

The 95% CI for sensitivity, specificity, and accuracy presented in Figure 7 are shown in Table 2. In Figure 7, the error bars in the accuracy and sensitivity statistics further complement this finding, providing insights into the variability and reliability of the diagnostic results. Specifically, the error bars for normal and malignant lesions were relatively narrow, indicating high consistency and reproducibility in the diagnostic outcomes. In contrast, the error bars for atypical hyperplasia were notably wider, reflecting greater variability in diagnostic results among different operators. Additionally, the error bars also reflect the stability of the TPM technology itself in diagnosing different lesions; the narrow error bars for normal and malignant lesions confirm that TPM can stably capture their characteristic features, while the wider error bars for atypical hyperplasia suggest that further optimization of TPM image interpretation standards or additional auxiliary diagnostic indicators may be needed to reduce diagnostic variability and improve the reliability of results for this challenging category. The highest accuracies were achieved for normal and malignant lesions, which present more distinct morphological features. Atypical hyperplasia demonstrated the lowest sensitivity, underscoring its status as a challenging diagnostic category due to its intermediate and often heterogeneous features, a known difficulty even in conventional pathology.

**Table 2:** Blinded diagnostic results of two pathologists

Tissue	Pathologist	Accuracy [95% CI]	Sensitivity [95% CI]	Specificity [95% CI]
Normal tissue	Pathologist 1	98.36% [96.02%, 99.42%]	90.91% [83.47%, 95.30%]	99.10% [97.18%, 99.73%]
	Pathologist 2	98.36% [96.02%, 99.42%]	90.91% [83.47%, 95.30%]	99.10% [97.18%, 99.73%]
Inflammatory tissue	Pathologist 1	84.43% [78.52%, 89.03%]	86.00% [77.56%, 91.78%]	83.33% [77.00%, 88.22%]
	Pathologist 2	84.43% [78.52%, 89.03%]	88.00% [79.80%, 93.20%]	81.94% [75.47%, 87.01%]
Intestinal metaplasia	Pathologist 1	95.90% [92.70%, 97.81%]	82.35% [73.17%, 88.93%]	98.10% [95.70%, 99.22%]
	Pathologist 2	90.98% [86.20%, 94.22%]	76.47% [66.47%, 84.23%]	93.33% [89.30%, 95.93%]
Atypical hyperplasia	Pathologist 1	86.89% [81.30%, 91.12%]	67.74% [56.37%, 77.23%]	93.41% [89.47%, 96.03%]
	Pathologist 2	86.89% [81.30%, 91.12%]	61.29% [49.97%, 71.43%]	95.60% [92.30%, 97.62%]
Malignant lesions	Pathologist 1	98.36% [96.02%, 99.42%]	92.31% [84.67%, 96.53%]	99.08% [97.13%, 99.72%]
	Pathologist 2	96.72% [93.50%, 98.52%]	76.92% [66.27%, 84.93%]	99.08% [97.13%, 99.72%]

### Quantitative collagen biomarkers for differentiating lesion risk

To develop objective, quantifiable biomarkers for distinguishing lesion severity and malignant potential, quantitative analysis of collagen fiber Area% and Dispersion was performed on SHG images. Tissues were categorized into Group A and Group B. The distributions of these parameters revealed striking differences.

Collagen fiber orientation dispersion: group B exhibited significantly higher dispersion (Median = 21.42, IQR [15.58-32.05]) compared to group a (Median = 13.38, IQR [8.38-17.94]). This indicates substantially more disordered, haphazardly arranged, and less organized collagen fibers in tissues with higher malignant potential (Mann-Whitney *U* = 684, *P* < 0.001, effect size *r* = −0.498).

Collagen fiber area percentage: group B also demonstrated a significantly higher area% (Median = 26.62%, IQR [24.15%-28.10%]) than group a (Median = 21.62%, IQR [19.85%-22.85%]). This points to an increased relative content of collagen, indicative of desmoplastic reactions and stromal remodeling, in tissues progressing towards or representing overt malignancy (Mann-Whitney *U* = 376, *P* < 0.001, effect size *r* = −0.644).

Sensitivity analysis: to further reduce statistical errors, we performed additional sensitivity analysis. For the sensitivity analysis, we calculated the mean values of all independent 3D stacks feature values derived from the same biopsy tissue, and then re-performed the Mann-Whitney *U* test on the averaged data to verify the robustness and reliability of the core results. The results are supplemented in Table 3 below: the comparison results after averaging can be obtained. The results indicate that the two indicators also have statistical significance when the statistical analysis is averaged at the biopsy level, and the *r* value after averaging has little deviation from the original *r* value. The correlation coefficients of %Area and Dispersion with the malignant degree of lesions are *r* = −0.62 and *r* = −0.53 (both *P* < 0.001), respectively. These values are highly consistent with the original effect sizes (*r* = −0.68 and *r* = −0.57), and there is no obvious change in the direction of correlation and statistical significance. This confirms that the original results are robust and not artificially exaggerated due to potential intra-patient correlation.

**Table 3:** Statistical analysis of collagen fiber indices, including results calculated at the ROI level and at the biopsy section level

Analysis		A	B	r	P
ROI analysis	*n*	78	44	–	–
	%Area	21.62 (19.85-22.85)	26.62 (24.15-28.10)	–0.644	<0.001
	Dispersion^o^	13.38 (8.38-17.94)	21.42 (15.58-32.05)	–0.498	<0.001
Biopsy analysis	*n*	40	20	–	–
	%Area	21.46 (19.63, 22.64)	26.65 (23.73, 28.37)	–0.680	<0.001
	Dispersion^o^	13.17 (10.50, 17.60)	23.05 (18.70, 27.36)	–0.507	<0.001

ROI: region of interest.

## Discussion

The most impactful finding of this research is the identification, quantification, and validation of alterations in collagen architecture. These alterations in collagen architecture are powerful biomarkers for distinguishing lesion severity. This finding has profound direct translational implications for clinical cancer diagnostics. Our quantitative analysis clearly shows that malignant lesions and atypical hyperplasia lesions are characterized by significantly increased collagen content (Area%) and a markedly more disorganized, haphazard collagen fiber network (Dispersion) compared to benign/low-risk conditions.^[23]^ This observation aligns with the established pathobiology of cancer, wherein tumor progression and invasion are often accompanied by extensive desmoplasia and remodeling of the ECM, which actively contributes to a tumor-permissive microenvironment.^[24, 25, 26]^ The capacity to objectively quantify these critical stromal alterations using SHG imaging provides powerful, data-driven parameters. These quantitative metrics are inherently less susceptible to subjective inter-observer variability than purely qualitative morphological assessments and are ideally suited for integration into machine learning algorithms. A key application of these quantitative parameters lies in investigating the remodeling of the ECM, which is primarily regulated by specific cellular subsets within the tumor microenvironment. Cancer-associated fibroblasts (CAF) are key cellular components in the tumor microenvironment. Tumor cells and CAFs can promote ECM remodeling by secreting ECM components and various proteases, thereby disrupting the integrity and stability of the ECM.^[27]^ During this process, the alignment and density of ECM fibers may be altered, leading to an increase in the orientation dispersion of the ECM. Moreover, mechanical stress in the tumor microenvironment exerts a profound influence on the structure and orientation of the ECM. CAFs generate mechanical forces through actin-myosin contraction, which act on ECM fibers, causing stretching, distortion, or rearrangement of the fibers. This consequently changes the orientation of ECM fibers and increases their orientation dispersion. In summary, increased orientation dispersion is likely the result of the combined effects of CAF-driven stromal remodeling, ECM degradation, and mechanical stress.^[28]^ These processes are intertwined and collectively modulate the structure and function of the ECM in the tumor microenvironment, facilitating tumor progression and metastasis. This paves the way for the development of automated, highly reproducible, and potentially more accurate diagnostic classification systems. These diagnostic classification systems could be transformative in clinical settings. In this study, the binary stratification that classifies intestinal metaplasia and chronic inflammation into the low-risk group, and dysplasia and malignant lesions into the high-risk group is clinically well-founded and fully consistent with the classic Correa cascade. Chronic inflammation and intestinal metaplasia represent early-stage precancerous lesions with low annual progression rates, and their progression can be blocked or even reversed with intervention. Therefore, assigning them to the low-risk group accords with the principles of clinical risk stratification. As a critical borderline lesion, dysplasia constitutes a key transitional stage in gastric carcinogenesis and, together with malignant lesions, falls into the high-risk category. Grouping these lesions allows precise discrimination between high-risk and low-risk individuals for gastric cancer. Thus, chronic inflammation and intestinal metaplasia are defined as low-risk lesions requiring routine monitoring, while dysplasia and malignant lesions are classified as high-risk lesions demanding intensive intervention. Regarding the influence of borderline lesions, especially dysplasia, on diagnostic performance, the sensitivity for dysplasia in this study was only 64.52%. This is closely associated with the pathological features and inherent diagnostic limitations of dysplasia: it exhibits multifocal distribution and high morphological heterogeneity. However, this sensitivity does not compromise the clinical rationality of the binary stratification. Given its high risk of progression, dysplasia should still be grouped with malignant lesions in the high-risk category. Diagnostic sensitivity for dysplasia can be further improved by standardizing biopsy procedures in future practice.

The diagnostic performance of TPM, while generally strong, varied across the different tissue categories. As anticipated, normal tissues and invasive malignant lesions, which exhibit the most distinct and contrasting morphological and stromal features, were diagnosed with high accuracy and specificity.^[26,29]^ The principal diagnostic challenge, as reflected by a lower mean sensitivity (64.52%), lay in the differentiation of atypical hyperplasia. This is not unexpected, as atypical hyperplasia represents a crucial but often subtle transitional state characterized by a spectrum of cytological and architectural atypia.^[30]^ These changes can be focal, heterogeneous, and frequently intermingled with inflammatory or metaplastic alterations, rendering its distinction inherently difficult even in conventional histopathology.^[25]^ Future research should focus on enhancing the TPM diagnosis of atypical hyperplasia. Our research should focus on incorporating more sophisticated quantitative morphometric or textural features. These features are derived from both TPEF signals and SHG signals. Examples of TPEF signal features include nuclear size distribution, nuclear texture, cell density, epithelial layer thickness, and gland-to-stroma ratio. Examples of SHG signal features include collagen fiber length, width, and curvature. This incorporation can potentially be achieved through advanced image processing techniques or AI-driven deep feature extraction. Analysis of larger, more diverse patient cohorts will also be essential for refining diagnostic algorithms for these challenging borderline lesions.^[31,32]^

Our study offers several important contributions that advance the field beyond previous TPM investigations in gastroenterology and gastric cancer. Firstly, we have provided a comprehensive characterization of TPM features across the full “normal to inflammation to intestinal metaplasia to atypical hyperplasia to cancer (malignant lesions)” continuum.^[33]^ This detailed mapping, with a particular focus on the often underrepresented but clinically pivotal precancerous states of intestinal metaplasia and atypical hyperplasia, is crucial for understanding the full diagnostic utility of TPM. Secondly, the rigorous validation of our TPM diagnostic features through a blinded review by independent, experienced pathologists significantly strengthens the credibility and potential clinical utility of the proposed criteria. Thirdly, we introduced dual quantitative collagen biomarkers (orientation dispersion and area percentage) and conducted robust statistical validation of these biomarkers. These biomarkers provide objective, measurable parameters for risk stratification. These parameters have not been extensively explored in this combined manner for the full spectrum of gastric lesions.^[34,35]^ Finally, our consistent use of cross-sectional imaging on fresh biopsies is more directly translatable to potential future endoscopic TPM applications for *in vivo* “optical biopsy”.^[36]^

The most compelling clinical advantage offered by the TPM approach described herein is its remarkable potential for speed and efficiency. TPM can furnish diagnostic-quality images and quantitative data from fresh biopsies within minutes of their acquisition. This rapid turnaround time has profound implications for transforming clinical practice. It could empower rapid on-site evaluation (ROSE) of endoscopic biopsy adequacy,^[37,38]^ potentially minimizing the need for repeat procedures and associated patient discomfort and costs. In a surgical oncology setting, TPM could facilitate near real-time intraoperative assessment of resection margins, potentially leading to more precise surgeries and reduced rates of local recurrence.^[22,39]^ In the future, we need to effectively integrate TPM technology into the existing workflow of the endoscopy center. The core of this integration is to select an appropriate application model based on clinical needs. It also involves balancing diagnostic efficacy and work efficiency while emphasizing translational relevance. The optimized integration plan combined with the characteristics of this study is as follows. First, the offline auxiliary tool model: gastric mucosal biopsy is performed during endoscopy, and the specimen is immediately sent to the pathology laboratory. Trained technicians complete TPM imaging and collagen orientation dispersion analysis to generate a standardized report. This model can serve as a supplement to routine pathological biopsy. It enables accurate screening of neoplastic lesions without disrupting the regular rhythm of endoscopic procedures, meeting the economic requirements of clinical translation. However, it is necessary to establish standardized expected operator training requirements for TPM interpretation. Based on previous experience, we have preliminarily defined the standardized operating procedures as follows. (1) Basics of two-photon pathology technology: the technical director explained the basic principles, imaging advantages (label-free, high resolution, deep imaging), image characteristics (collagen fiber autofluorescence, clear cell morphology) of TPM, and its core differences from traditional H&E-stained sections (no staining required, observable *in situ* morphology of living/fresh tissues). (2) Pathological basis of gastric mucosal lesions and TPM image diagnostic standards: pathologists explained the pathological characteristics of normal gastric mucosa, chronic inflammation, intestinal metaplasia, atypical hyperplasia and gastric adenomalignant lesions (combined with our diagnostic standards) as well as their typical TPM manifestations, clarified diagnostic and differential points, and emphasized the difficulties in differentiating atypical hyperplasia from chronic inflammation and malignant lesions. (3) Practical operation: pathologists observed training sample TPM images separately, compared with H&E-stained sections, identified lesion morphological characteristics and recorded diagnostic results; trainers provided on-site guidance, explained existing problems and corrected common errors in collagen fiber identification and lesion grading. (4) Case review: summary and review of typical cases and cases with diagnostic differences during training. (5) Assessment: theoretical and practical examinations to quantify training outcomes. Based on the above, we aim to train relevant technical personnel who are proficient in both the imaging characteristics of TPM and the operating procedures, so as to achieve technological integration and better apply TPM technology in clinical practice.

Second, the real-time decision-making tool model: in subsequent studies, the TPM imaging module can be integrated with endoscopic equipment to achieve a closed-loop workflow of “endoscopic examination-real-time imaging-instant diagnosis”. During endoscopy, once suspicious lesions are identified, the endoscopist directly performs *in vivo* imaging on the lesion site using a TPM imaging probe mounted at the distal end of the endoscope. The total duration for mosaic imaging and 3D stack imaging is controlled within 10 min. This procedure enables real-time acquisition of data regarding collagen fiber orientation dispersion as well as lesion risk stratification results. Looking further into the future, continuous development of miniaturized TPM probes compatible with conventional endoscopes shows great potential. Such probes enable direct *in vivo* “optical biopsy” during endoscopic procedures. Accordingly, they are expected to revolutionize the screening and surveillance of gastric cancers. This could allow for immediate lesion characterization, guide more targeted physical biopsies to areas of highest suspicion, or, in some instances of clearly benign or low-risk lesions, even obviate the need for physical biopsy altogether, leading to less invasive and more efficient patient care.

This study, while demonstrating significant promise, has certain limitations that warrant acknowledgment. Despite employing fresh human tissue specimens, our *ex vivo* experimental setup fails to fully recapitulate the dynamic complexity and motion artifacts inherent to *in vivo* imaging conditions. In addition, the sample size in this study was relatively limited, especially for normal tissue and overtly malignant groups. Further validation in larger prospective multi-center cohorts is therefore warranted to verify the robustness of the present findings, optimize the diagnostic thresholds of quantitative biomarkers, and evaluate the generalizability of our method across different patient populations and imaging devices. While our cross-sectional imaging strategy shows high relevance for future endoscopic applications, we acknowledge its inherent limitations. This approach may not fully characterize certain pathological conditions such as atrophic gastritis, in which glandular volume loss represents a key feature. Such structural alterations may be more accurately evaluated using elaborate 3D reconstruction and volumetric analysis. Admittedly, the inherent anatomical characteristics of the gastric lumen—featuring an extensive surface area with abundant, irregularly distributed mucosal folds—inevitably lead to localized sampling limitations in gastric biopsy specimens. Reliance solely on single or sparse biopsy markers for disease progression surveillance carries inherent diagnostic risks. Nevertheless, TPM imaging of biopsy-derived gastric mucosal tissue remains of irreplaceable significance. TPM allows for high-resolution visualization of key three-dimensional microarchitectural features, including the 3D structure of gastric pits and the spatial distribution of collagen fibrils, thereby effectively compensating for the localized sampling bias caused by the irregular mucosal folds of the gastric cavity.

Future research will concentrate on several critical directions to advance the present work. First, we aim to enlarge the patient dataset across all lesion categories, develop sophisticated artificial intelligence and machine learning algorithms for automated image classification and feature extraction from TPM data, and explore additional quantitative parameters derived from TPEF and SHG signals to further improve diagnostic accuracy, especially for borderline lesions such as atypical hyperplasia. Second, efforts will be actively devoted to technological innovations required for the clinical translation of TPM techniques into routine *in vivo* endoscopic applications.

## Conclusion

In conclusion, TPM, particularly when combined with quantitative analysis of collagen architecture, is a promising technological approach for the diagnosis of gastric lesions. It provides a rapid, label-free method for visualizing key pathological features in fresh, unprocessed tissues and offers objective, quantifiable biomarkers for differentiating benign lesions from malignant lesions and atypical hyperplasia lesions. This study provides a critical and robust foundation for the future development and clinical integration of TPM-based diagnostic systems. Such systems, potentially augmented by artificial intelligence, may accelerate diagnostic workflows, improve diagnostic accuracy, enable earlier and more precise interventions, and enhance the management and outcomes for patients with gastric malignant lesions and precursor lesions.
